# Integrated proteomics and metabolomics reveals the comprehensive characterization of antitumor mechanism underlying Shikonin on colon cancer patient-derived xenograft model

**DOI:** 10.1038/s41598-020-71116-5

**Published:** 2020-08-24

**Authors:** Yang Chen, Juan Ni, Yun Gao, Jinghui Zhang, Xuesong Liu, Yong Chen, Zhongjian Chen, Yongjiang Wu

**Affiliations:** 1 College of Pharmaceutical Sciences, Zhejiang University, Hangzhou, 310058 China; 2 Institute of Cancer and Basic Medicine (ICBM), Chinese Academy of Sciences, Zhejiang Province, Hangzhou, 310022 China; 3 Cancer Hospital of the University of Chinese Academy of Sciences, Zhejiang Province, Hangzhou, 310022 China; 4 Zhejiang Cancer Hospital, Zhejiang Province, Hangzhou, 310022 China

**Keywords:** Cancer metabolism, Pharmacology

## Abstract

Colorectal cancer (CRC) is a common malignancy occurring in the digestive system. Despite progress in surgery and therapy options, CRC is still a considerable cause of cancer mortality worldwide. In this study, a colon cancer patient-derived xenograft model was established to evaluate the antitumor activity of Shikonin. The protective effect underlying Shikonin was determined through assessing serum levels of liver enzymes (ALT, AST) and kidney functions (BuN, Scr) in PDX mice. Proteomics and metabolomics profiles were integrated to provide a systematic perspective in dynamic changes of proteins and global endogenous metabolites as well as their perturbed pathways. A total of 456 differently expressed proteins (DEPs), 32 differently expressed metabolites (DEMs) in tumor tissue, and 20 DEMs in mice serum were identified. The perturbation of arginine biosynthesis, purine metabolism, and biosynthesis of amino acids may mainly account for therapeutic mechanism of Shikonin. Furthermore, the expression of mRNAs participating in arginine biosynthesis (CPS1, OTC, Arg1) and do novo purine synthesis (GART, PAICS, ATIC) were validated through RT-qPCR. Our study provides new insights into the drug therapeutic strategies and a better understanding of antitumor mechanisms that might be valuable for further studies on Shikonin in the clinical treatment of colorectal cancer.

## Introduction

Colorectal cancer (CRC), a digestive system tumor, is currently the third most common malignant cancer worldwide^1^. Despite the improvements in diagnosis and therapeutic methods, the high risk of liver metastasis and poor prognosis of patients with advanced colorectal cancer remain to be addressed^2^. Currently, surgery combined with chemotherapy and radiotherapy is still served as the predominant treatment for colorectal cancer. However, their side effects are considerable. Hence, the development of natural products has become a priority for the therapy of colorectal cancer.

**Figure 1:**
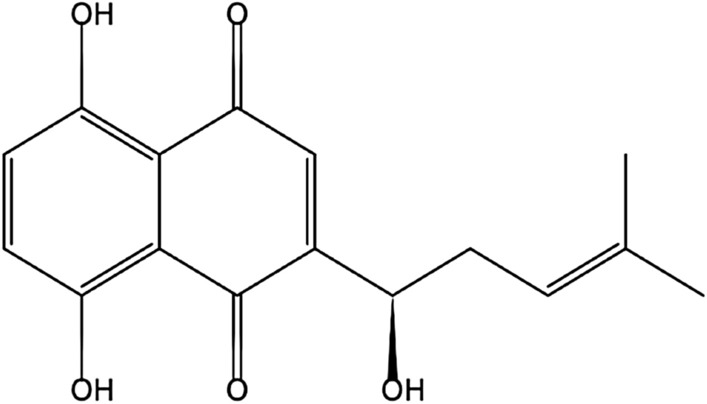
The structure of Shikonin.

Shikonin (SHK), a naphthoquinone pigment isolated from the root of *Lithospermum erythrorhizon* (Sieb. et Zucc, Boraginaceae) (Fig. 1), has been reported to be highly effective against a variety of cancer types during the past decades^3^. Shikonin was demonstrated to suppress proliferation and induce cell cycle arrest in human colon cancer through inhibiting hypoxia-inducible factor-1 alpha signaling both in vitro and vivo^4^. Shikonin induced apoptosis of paclitaxel-resistant non-small cell lung cancer (NSCLC) cell lines and xenograft tumors through suppressing NEAT1 and Akt signaling^5^. Recently, Shikonin was reported to impede biosynthetic pathways critical for leukemia cell survival by the combination of dimethylaminoparthenolide^6^. Considering the excellent antitumor activity of Shikonin, it is imperative to discover its molecular pharmacological mechanisms.

In our current study, a colorectal cancer patient-derived xenograft (PDX) model was generated to determine the antitumor effect of Shikonin on tumor growth and intratumoral metabolomics alterations. Compared with the majority of xenograft models based on immortalized cancer cell lines engrafted in mice, tumor samples from clinical patients rapidly grafted into immunodeficient mice to develop PDXs provide a more clinically applicable strategy for potential therapeutic study^7^. Moreover, omics techniques with advanced analysis tools have widely utilized in medical diagnostics and basic research, including characterizing complex biosystems and illuminating the therapeutic mechanisms in various diseases^8^. Proteomics is mainly performed to identify the de-regulated dynamic proteins and their interactions in body fluids which could provide a deep understanding of the biological system at the protein level^9^, while metabolomics is extensively applied to comprehensively evaluate dynamic changes of global endogenous metabolites and their perturbed pathways^10^. The integration of multi-omics profiles may provide a better understanding of the drug therapeutic strategies and higher reliability in illustrating the treatment effects and mechanisms^11^. Previous studies on Shikonin were mostly limited to reveal the antitumor mechanism and molecular targets though traditional biochemical methods, the systematic effect of Shikonin remains poorly understood.

In the present study, an established PDX approach coupled with tandem mass tag (TMT)-based quantitative proteomic analysis and untargeted LC–MS-based metabolomics analysis was utilized to investigate molecular alterations under Sikonin treatment. Compared with the previous study, our study provided the first characterization of the effect of Shikonin on PDX tumor growth and gain comprehensive insights into the mechanisms and molecular response regarding Shikonin to explore the possible signatures.

## Results

### Effect on body weight and tumor volume

**Figure 2:**
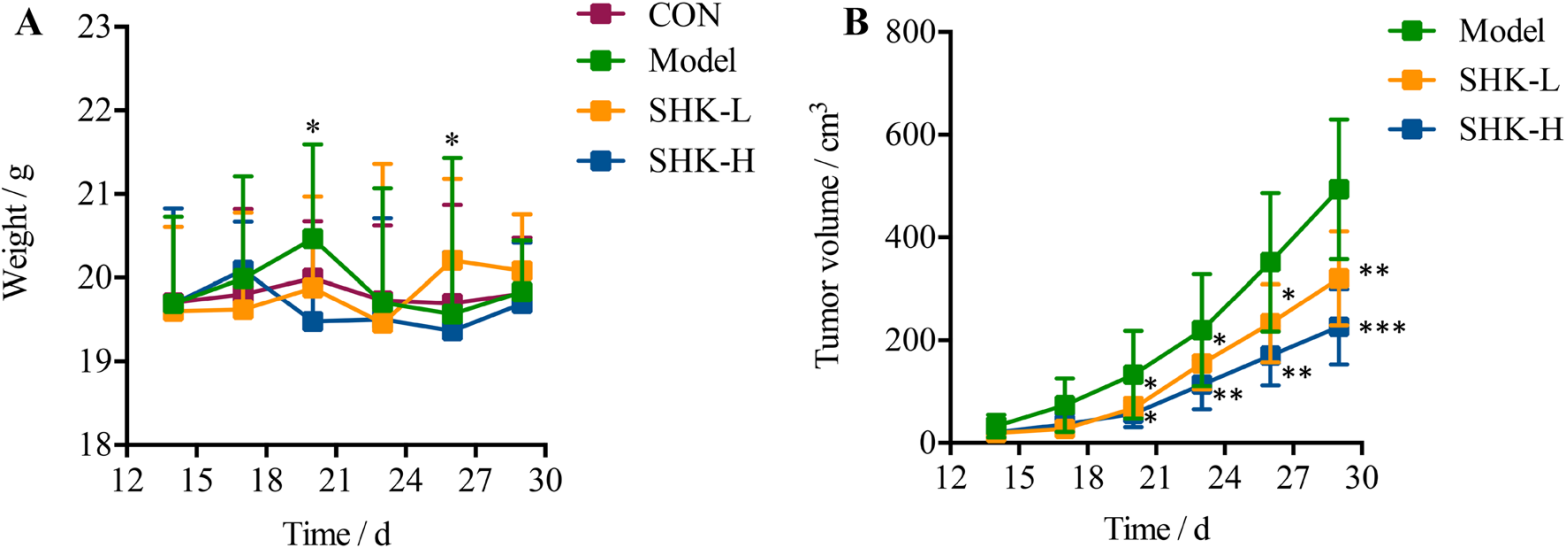
Effect of administration of Shikonin on PDX mice. A. Body weight changes of mice among control, model, and Shikonin-treated groups. B. Tumor inhibitory rate of Shikonin in PDX mice ($\overline{x}$ ± *s*, *n* = 10). *p < 0.05; **p < 0.01; ***p < 0.001.

The body weight and tumor volume of mice were measured every three days after administration. Overall, the bodyweight of all groups remained at a stable level during the trail and a slight frustration was observed at Day 20 and 26, respectively (Fig. 2A). Compared with the model group, mice in Shikonin-treated groups showed a significant reduction in tumor volume after the treatment for a week (*p* < 0.05). With the administration time prolonging, the difference between the Shikonin-treated group and model group increased continuously and the tumor inhibitory rate of Shikonin was 23.87% and 41.85% finally when administered at 1 mg/kg or 2 mg/kg (Fig. 2B). Besides, mice under high dosage treatment induced a highly significant reduction of tumor volume (*p* < 0.001) in a shorter time compared with the low dosage group, which indicated the antitumor effect of Shikonin in a dose-dependent manner. These data demonstrated that Shikonin remarkably inhibited the tumor growth in colon cancer PDX mice.

### Shikonin alleviates liver and kidney dysfunction in PDX models

**Figure 3:**
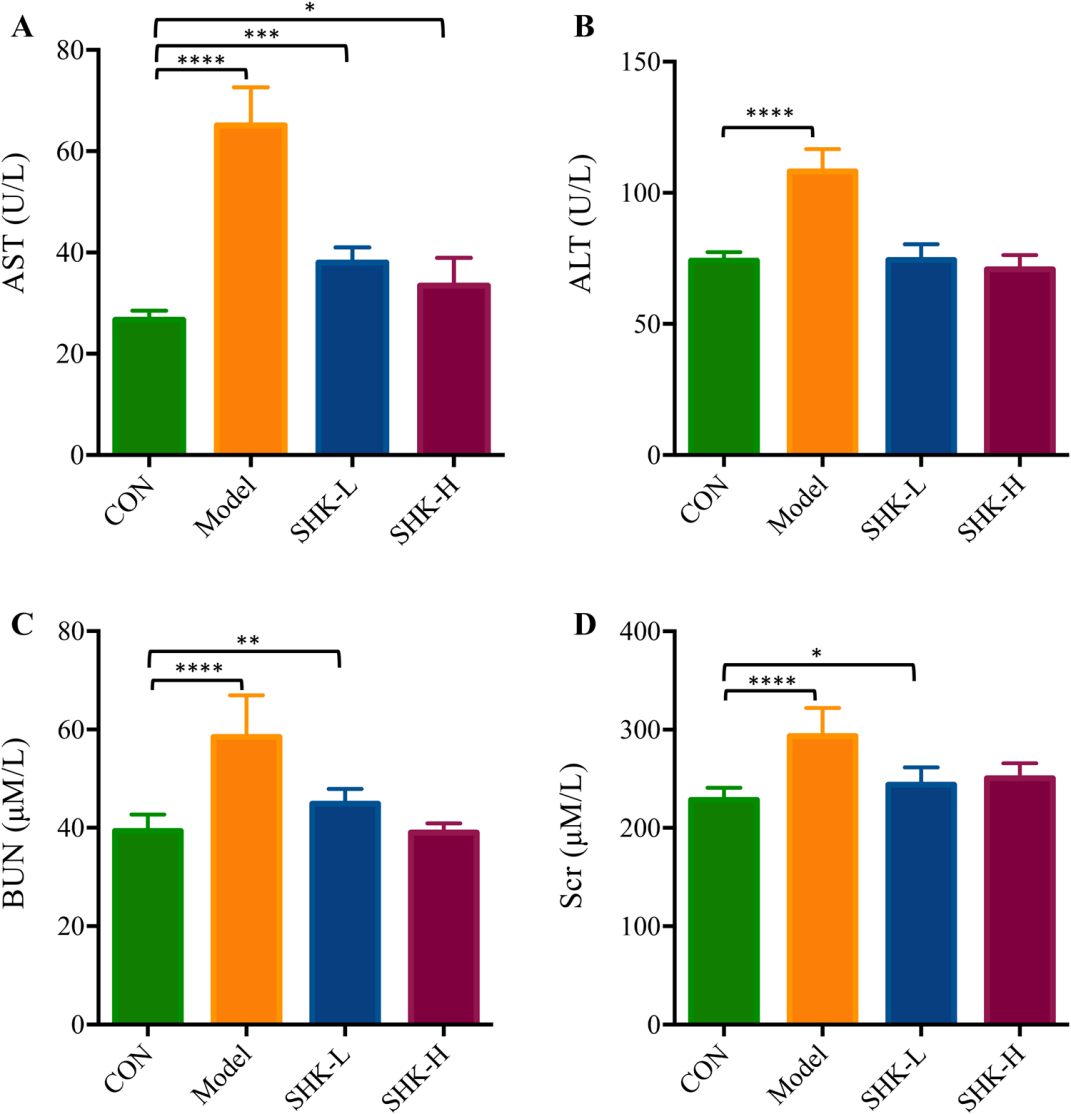
Effects of Shikonin on liver enzymes (ALT, AST) and kidney functions (BuN, Scr) in PDX mice. Each bar with vertical line represents the mean ± SEM of ten mice per group. *p < 0.05; **p < 0.01; ***p < 0.001; ****p < 0.0001 versus control group only.

To evaluate the acute toxicity of Shikonin in vivo, the activity of alanine transaminase (ALT), aspartate transaminase (AST), serum creatinine (Scr), and blood urea nitrogen (BUN) were measured. As presented in Fig. 3, the levels of AST, ALT, BUN, and Scr were up-regulated significantly after tumor xenograft, while the expression in serum with a reversing trend to normal induced by Shikonin treatment compared with those in the control group. The results suggested that Shikonin could alleviate the liver and kidney dysfunction in tumor xenograft mice.

### Differentially expressed proteins

**Figure 4:**
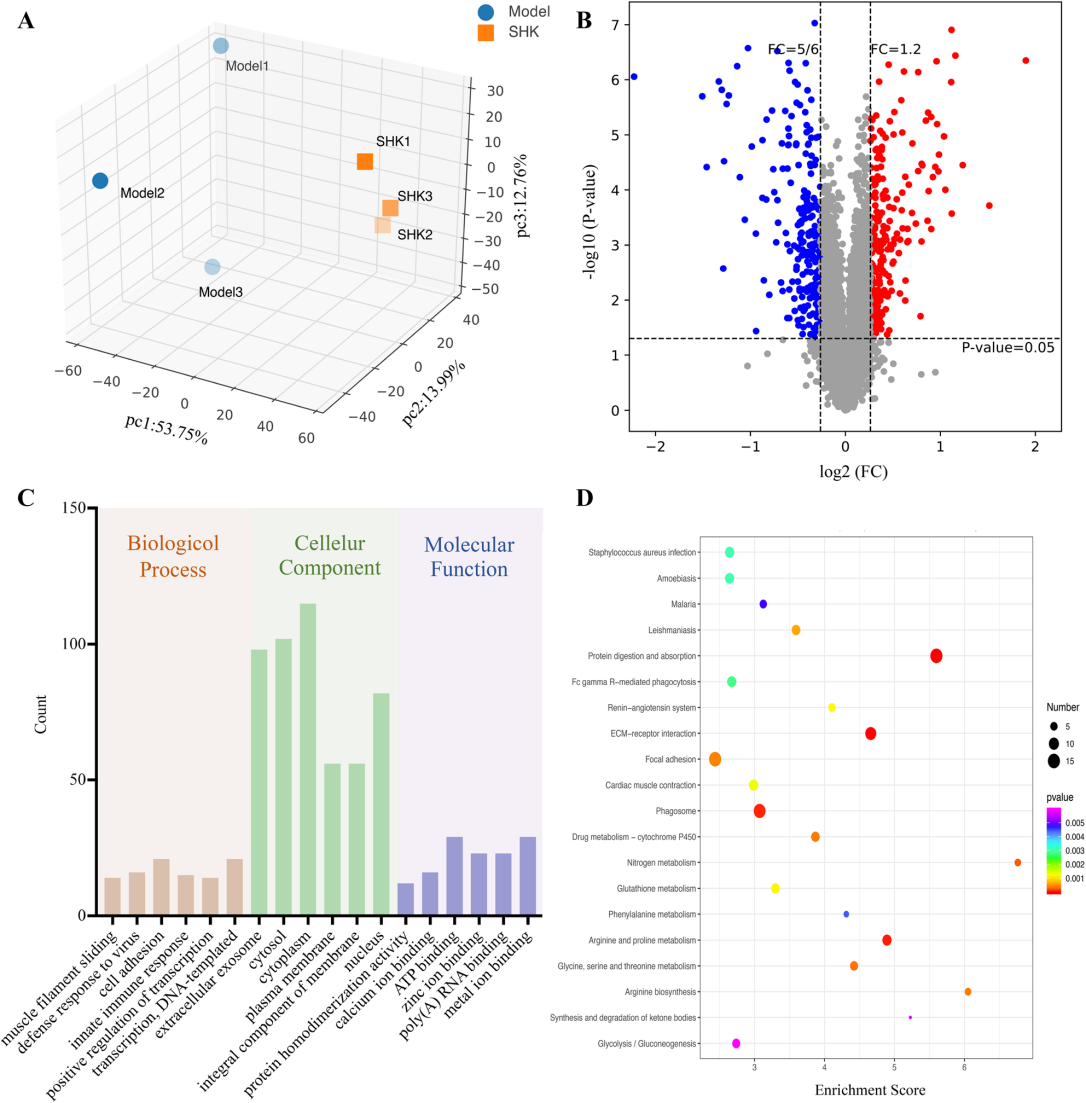
Tumor tissue proteomics analysis for PDX mice under the treatment of Shikonin. (**A**) PCA score plots of DEPs from the model group and Shikonin-treated group (n = 3). (**B**) Volcano plots analysis of DEPs. (**C**) GO enrichment analysis of DEPs. (**D**) KEGG analysis of DEPs (Top 20).

To reveal the potential mechanism of Shikonin in colon cancer PDX mice, the protein expression was compared between the model group and Shikonin-treated groups. Unsupervised pattern recognition was used in data analysis. As shown in Fig. 4A, obvious separation trend between the model and Shikonin-treated group was visualized in the PCA score plot. A total of 256 proteins including 140 up-regulated proteins and 116 down-regulated proteins in the Shikonin-treated group were identified as the differentially expressed proteins (DEPs) that were possibly involved in molecular targets. The volcano plot was also presented based on the threshold value of fold change (< 0.8 or > 1.2) and *p* value (< 0.05) (Fig. 4B).

### Bioinformatics analysis

**Figure 5:**
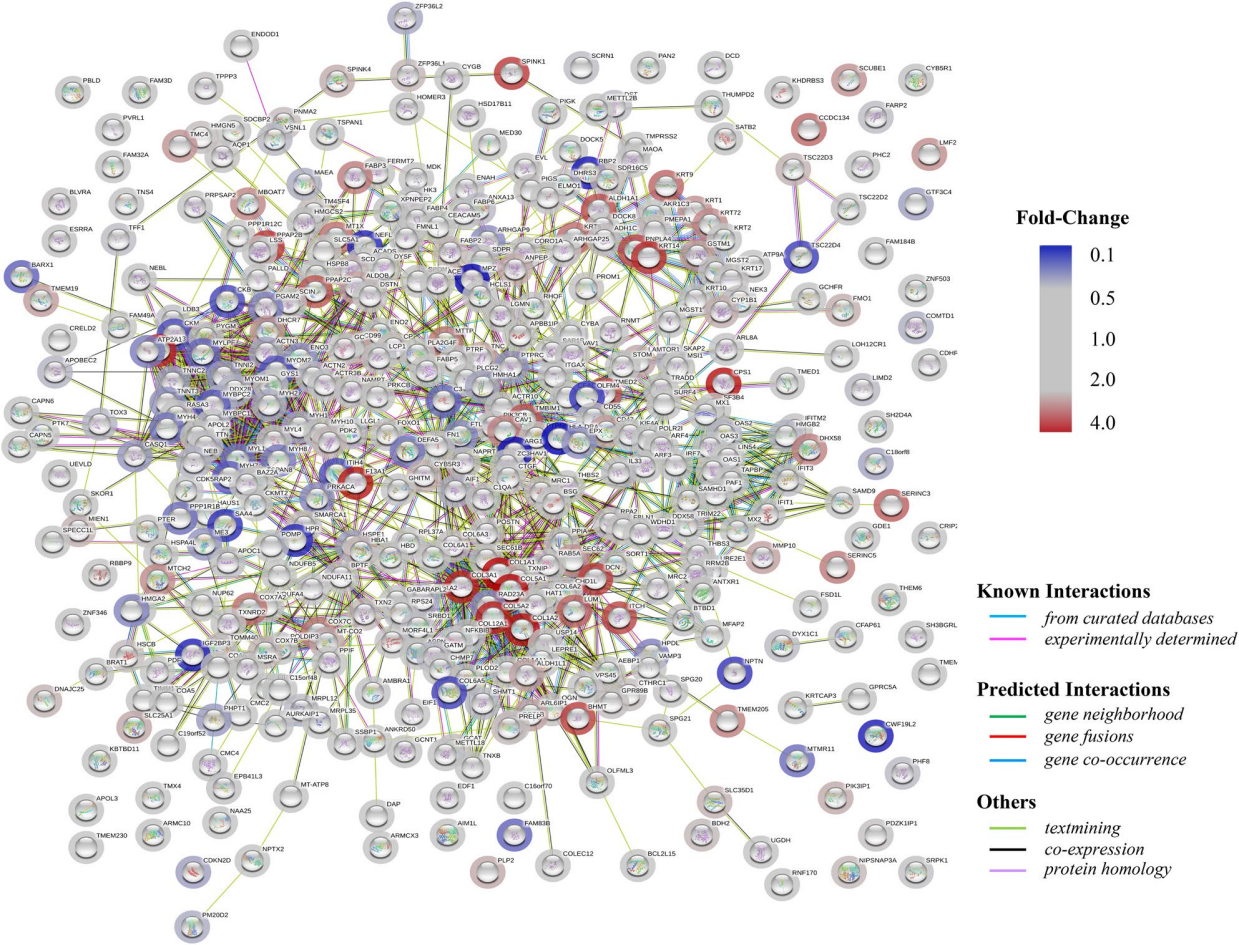
Protein–protein interaction (PPI) networks analysis of the DEPs. (Circles: DEPs; Straight lines: the interactions between different proteins; Colors: type of evidence for the connection).

GO enrichment analysis was performed to cluster the cellular components, molecular functions and biological processes (Fig. 4C). The biological process analysis indicated that DEPs mainly involved in muscle filament sliding, defense response to virus, cell adhesion, innate immune response, positive regulation of transcription, DNA-templated and transcription DNA-templated. The molecular functions revealed that DEPs played important roles in protein homodimerization activity, calcium ion binding, ATP binding, zinc ion binding, poly(A) RNA binding and metal ion binding. The cellular component analysis suggested that DEPs mostly belonged to extracellular exosome, cytosol, cytoplasm, plasma membrane, integral component of membrane and nucleus. Meanwhile, KEGG was also used for understanding high-level functions and utilities of the biological system. The top 20 relevant pathways were shown in Fig. 4D. From KEGG pathway analysis, these proteins were enriched in protein digestion and absorption, ECM-receptor interaction, focal adhesion, glycine, serine and threonine metabolism and arginine and proline metabolism mostly. Besides, as presented in Fig. 5, the PPI networks associated with the DEPs were generated through the STRING database. Also, the cut-off value of FC was further set as > 2 and < 0.5 to select the proteins. In total, 13 DEPs were screened and shown in Table 1. To better visualize and characterize therapeutic effect of Shikonin, the heatmap of DEPs in PDX mice with high-dosage of Shikonin and the model group was shown in Fig. S1A. The PPI networks involved in 13 DEPs were mainly related to arginine biosynthesis and biosynthesis of amino acids (Fig. S1B).

**Table 1:** The proteins identified as differentially expressed with TMT-labeling proteomics.

No	Protein name	Gene name	Coverage (%)	Unique peptides	MW (kDa)	FC	*p* value	SHK versus model
P1	Arginase-1	ARG1	7	2	34.7	0.4	4.78E−05	Down
P2	Collagen alpha-1(I) chain	COL1A1	13	16	138.9	2.87	3.10E−05	Up
P3	Collagen alpha-2(I) chain	COL1A2	3	4	129.2	2.28	6.49E−07	Up
P4	Collagen alpha-1(III) chain	COL3A1	4	6	138.5	2.06	6.46E−06	Up
P5	Collagen alpha-1(V) chain	COL5A1	1	2	183.4	2.53	1.86E−07	Up
P6	Collagen alpha-2(V) chain	COL5A2	1	2	144.8	2.17	9.58E−06	Up
P7	Mast cell carboxypeptidase A	CPA3	5	2	48.6	2.03	3.27E−05	Up
P8	Carbamoyl-phosphate synthase [ammonia], mitochondrial	CPS1	4	5	164.8	2.37	5.04E−05	Up
P9	Coagulation factor XIII A chain	F13A1	3	2	83.2	2.27	8.11E−03	Up
P10	Histone H1.1	H1-1	20	1	21.8	0.29	7.83E−03	Down
P11	HLA class II histocompatibility antigen, DR alpha chain	HLA-DRA	5	1	28.6	0.38	2.93E−06	Down
P12	Immunoglobulin lambda variable 4–60	IGLV4-60	8	1	13	4.68	7.56E−05	Up
P13	Myelin protein P0	MPZ	8	2	27.5	0.38	8.00E−07	Down

### Tumor tissue metabolomics and serum metabolomics analysis

**Figure 6:**
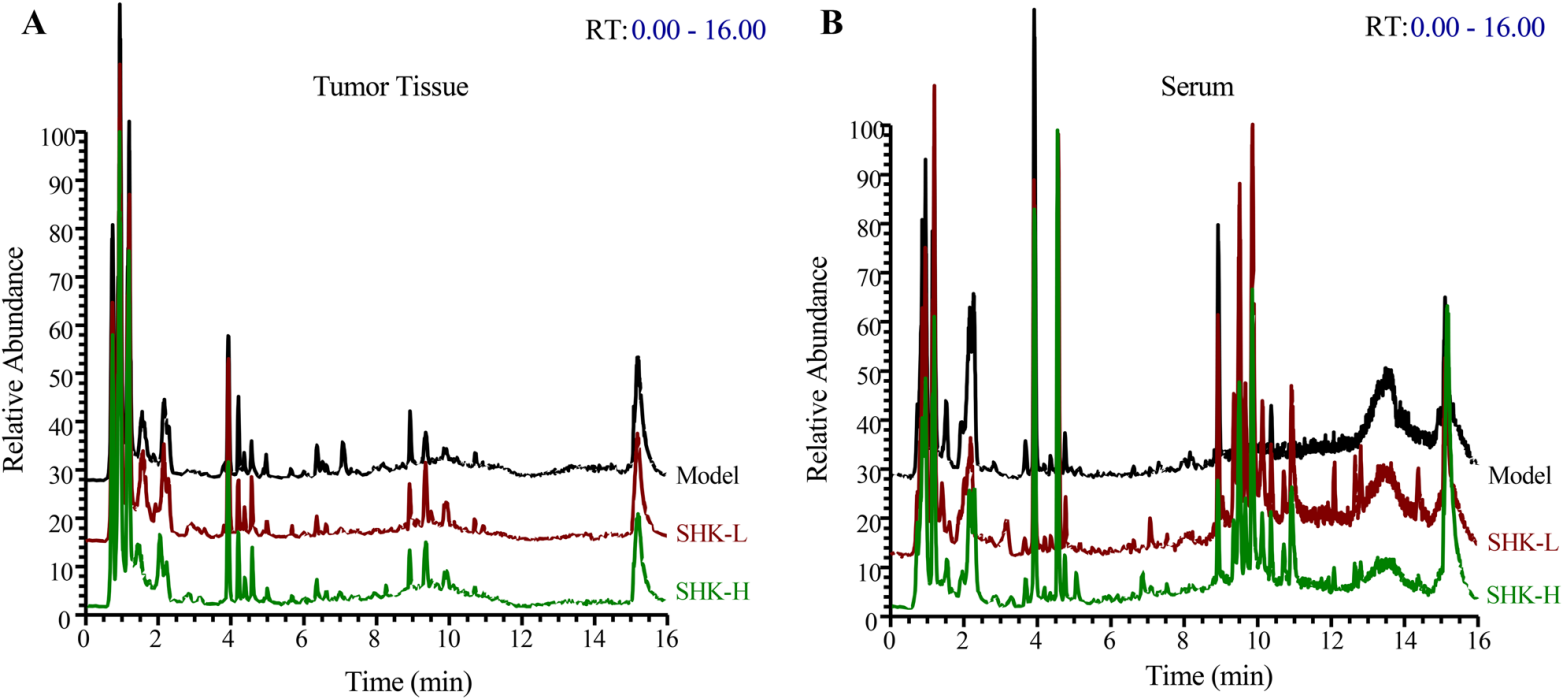
Typical total ion chromatograms (TIC) from PDX mice tumor tissue (**A**) and serum (**B**) samples in positive ion modes.

**Figure 7:**
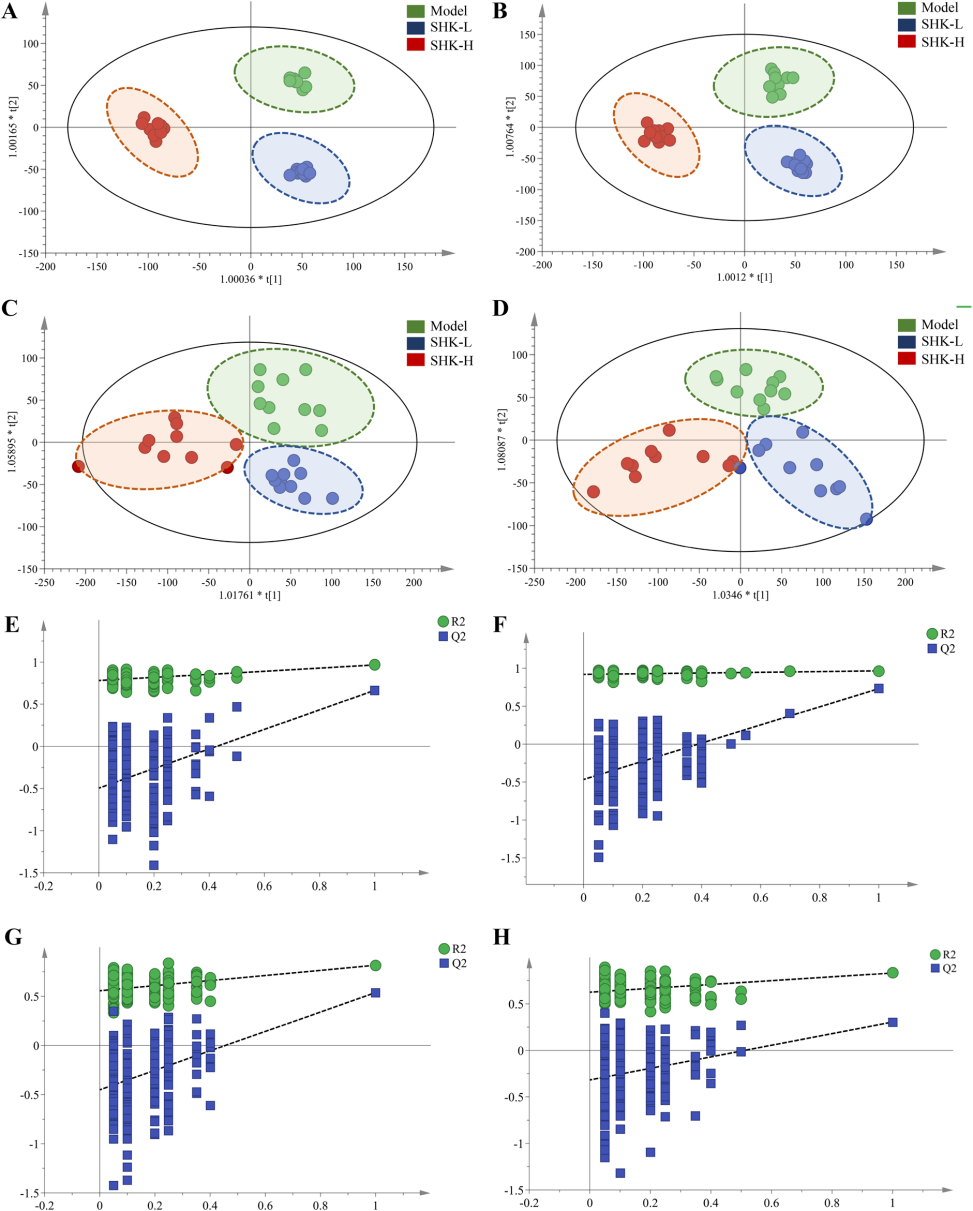
OPLS-DA score plots and the permutation tests for OPLS-DA models of PDX mice tumor tissue and serum metabolomics. OPLS-DA score plots of mice tumor tissue metabolites in positive (**A**) and negative ion mode (**B**). OPLS-DA score plots of mice serum metabolites in positive (**C**) and negative ion mode (**D**). Permutation tests for OPLS-DA models of mice tumor tissue metabolites in positive (**E**) and negative (**F**) ion mode. Permutation tests for OPLS-DA models of mice serum metabolites in positive (**G**) and negative (**H**) ion mode.

To further reveal the perturbation of Shikonin therapeutic intervention on colon cancer PDX mice from a comprehensive perspective, both tumor tissue and serum metabolomics were performed. Typical total ion chromatograms (TIC) from PDX mice tumor tissue and serum samples in positive ion modes were shown in Fig. 6. To obtain the difference of metabolic components among the model, low-dosage Shikonin treatment, and high-dosage Shikonin treatment groups, multivariate statistical analysis method was applied to obtain information for sorting and identifying metabolites. The metabolomic profiles in three groups were evaluated with OPLS-DA score plots in positive and negative mode, which presented the best separation of model and drug experimental groups. The cross-test parameters R^2^X, R^2^Y, and Q^2^ values of OPLS-DA model from PDX mice tumor tissue metabolites were 0.557, 0.974, 0.749 and 0.562, 0.965, 0.773 in positive and negative mode (Fig. 7A,B), while the cross-test parameters R^2^X, R^2^Y, and Q^2^ values of OPLS-DA model from PDX mice serum metabolites were 0.422, 0.790, 0.513 and 0.503, 0.804, 0.532 in positive and negative mode (Fig. 7C,D), which suggested the good fitness and prediction of the established model. Furthermore, the R^2^Y-intercept of tissue metabolites was 0.791 and 0.932 in the positive and negative ion modes (Fig. 7E,F). And the R^2^Y-intercept of serum metabolites was 0.491 and 0.532 in the positive and negative ion modes (Fig. 7G,H), respectively. Besides, all of R2 values in green were lower than the original points to the right which suggested the validation of the original model.

### Identification of biomarkers and pathways

**Figure 8:**
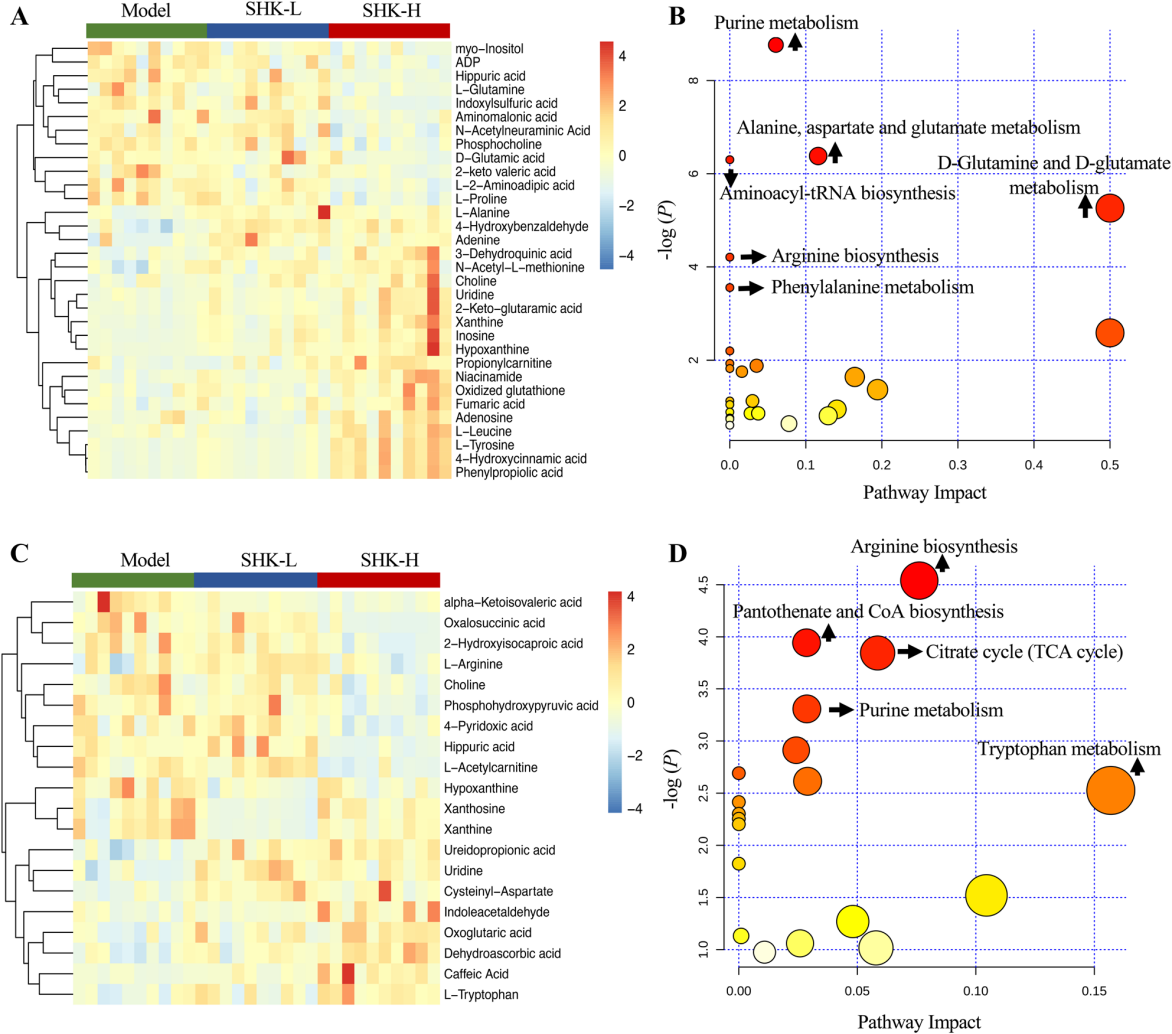
Heatmap and pathway analysis of DEMs from mice metabolic analysis. Heatmap (**A**) and pathway enrichment (**B**) of 32 DEMs from mice tumor tissue metabolic analysis. Heatmap (**C**) and pathway enrichment (**D**) of 20 DEMs from mice serum metabolic analysis.

**Figure 9:**
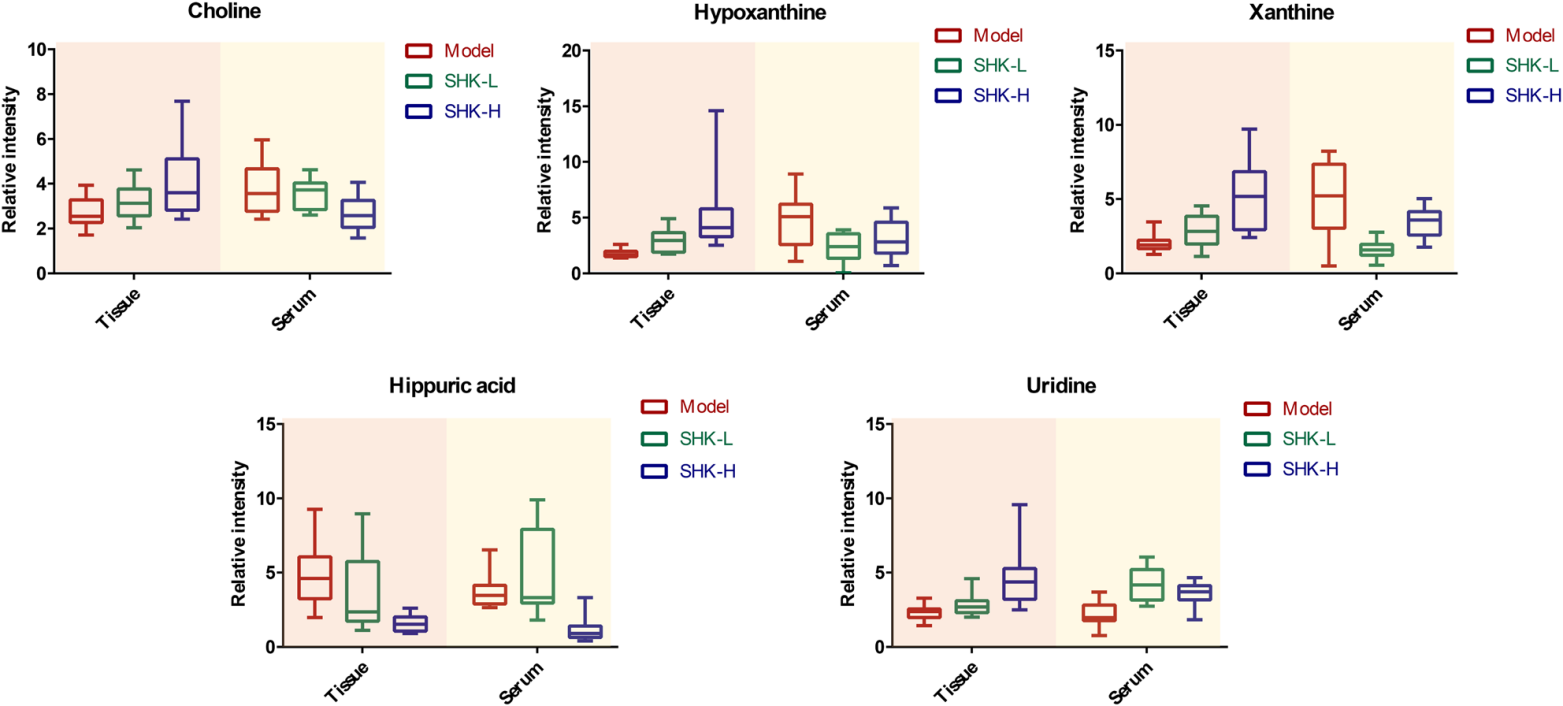
The changes of common metabolites in PDX mice tumor tissue and serum from the model, low-dosage Shikonin treatment (SHK-L) and high-dosage Shikonin treatment (SHK-H) groups.

To identify differently expressed metabolites (DEMs) in the model and Shikonin-treated groups, the variables with VIP values (based on OPLS-DA) ≥ 1 and *p* value ≤ 0.05 (one-way ANOVA analysis) were filtered out. The screened DEMs were subsequently identified according to MS/MS fragment matched with online database. Finally, a total of 32 DEMs from mice tumor tissue metabolomics and 20 DEMs from mice serum metabolomics were identified. These metabolites might account for the antitumor activity of Shikonin on colon cancer PDX mice. As presented in Table S1, twenty of 32 DEMs from mice tumor tissue identified were up-regulated and twelve of them decreased after being treated with Shikonin. Similarly, Table S2 showed that eight of the 20 DEMs from mice serum identified increased and twelve of them decreased compared with the model group. To better visualize the DEMs involved in therapeutic effect of Shikonin, a heatmap analysis was performed. Based on distribution of colors, the metabolism of Shikonin-treated groups showed the significant change compared with model groups both in mice tissue and serum, especially the groups with high-dosage treatment (Fig. 8A,C). To further excavate the perturbation influenced by DEMs, the metabolic pathways were analyzed through MetaboAnalyst 4.0. Many markedly changed pathways in the model and Shikonin-treated groups were discovered at the metabolomic level. The pathways influenced by DEMs from mice tumor tissue metabolites were involved in purine metabolism, alanine, aspartate and glutamate metabolism, aminoacyl-tRNA biosynthesis, arginine biosynthesis, and D-Glutamine and D-glutamate metabolism (Fig. 8B). In addition, pathways including arginine biosynthesis, pantothenate and CoA biosynthesis, citrate cycle (TCA cycle) and purine metabolism were perturbed from mice serum metabolic analysis (Fig. 8D). In particular, five DEMs were identified both in mice tumor tissue and serum, including xanthine, hypoxanthine, uridine, choline and hippuric acid. As shown in Fig. 9, xanthine, hypoxanthine and choline increased in tumor tissue while decreased in mice serum, which indicated that they probably transferring from the serum into tumor tissue after Shikonin treatment. Meanwhile, with the increase of dosage, the deviation became more significant, suggesting that the degree of therapeutic effect was positively correlated with the dosage. Besides, uridine and hippuric acid which showed the same tendency in both tumor tissue and serum implied the changes in the total expression of PDX mice after treatment.

### Integrated network analysis and RT-qPCR validation

**Figure 10:**
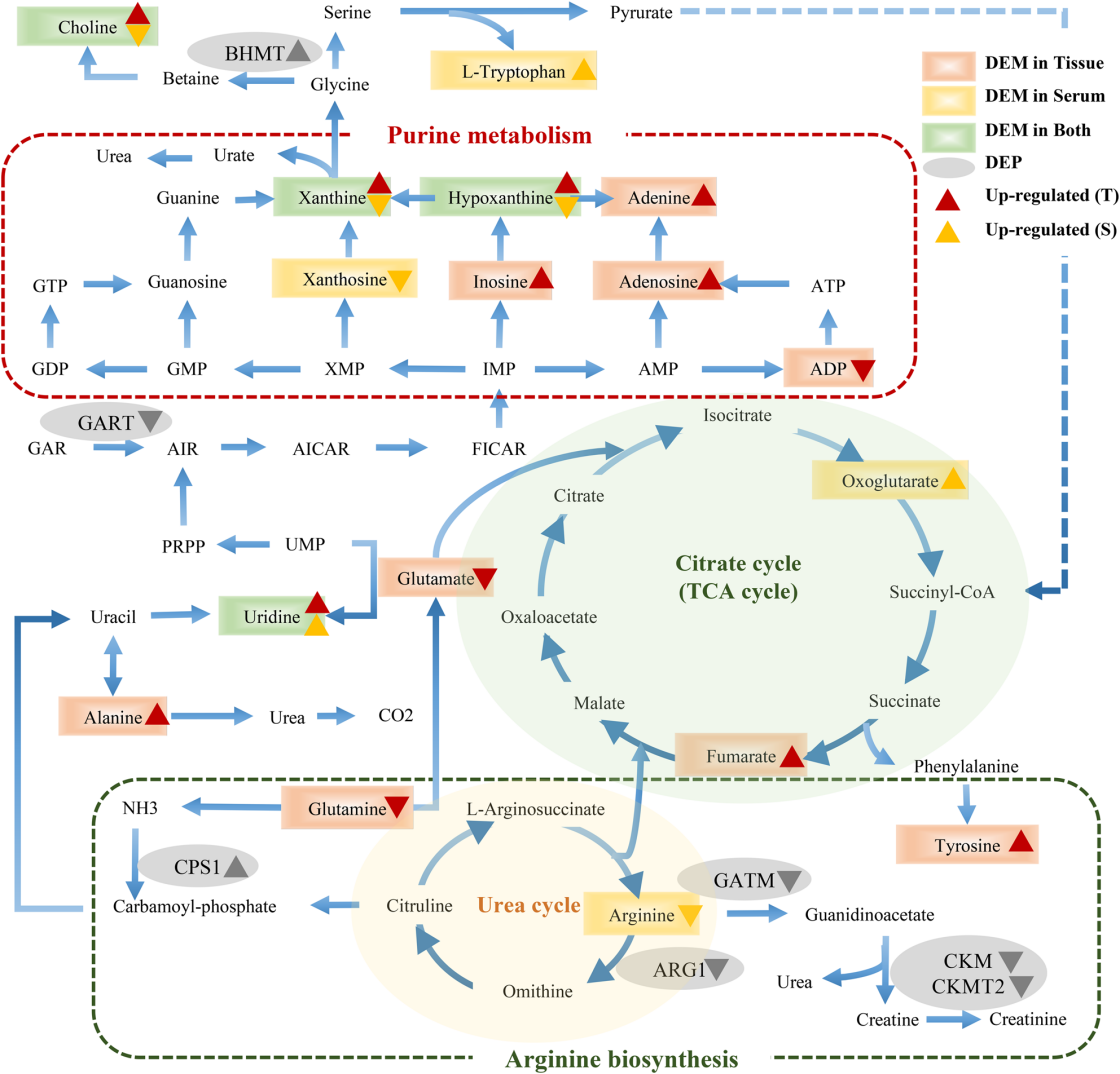
The perturbed proteins and metabolites corresponding metabolic pathways related to Shikonin treatment by integrating the proteome and metabolome data sets.

**Figure 11:**
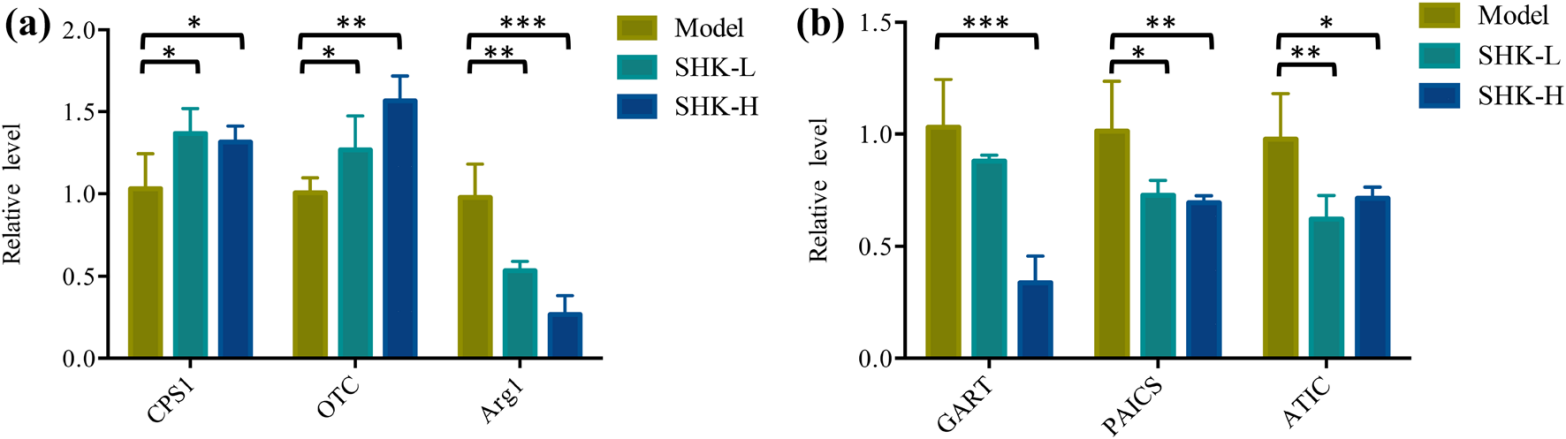
The expression level of mRNAs involved in arginine biosynthesis and do novo purine synthesis in tumor tissues. (**A**) Relative level of arginine biosynthesis-related mRNAs. (**B**) Relative level of mRNAs in do novo purine synthesis.

To better provide a comprehensive understanding of biological systems in response to Shikonin treatment, proteomics and metabolomics data were integrated to acquire the shared pathways and interaction between DEPs and DEMs. The systematic scheme of regulated metabolites and related proteins under Shikonin treatment were summarized (Fig. 10). The results indicated that the disorder of arginine biosynthesis, purine metabolism, biosynthesis of amino acids, and glutathione metabolism were involved in antitumor effect of Shikonin. In particular, the perturbation of arginine biosynthesis revealing an obvious differentiation in proteomics profile and metabolomics profiles from mice tumor tissue and serum was inferred as the major pathway associated with Shikonin therapeutic effect. Meanwhile, the expression of Cps1, OTC, and Arg1 joint in arginine biosynthesis and GART, AICS, and ATIC involved in do novo purine synthesis pathway were further assessed though RT-qPCR. Cps1 and OTC serving as function of urea cycle enzymes were up-regulated and Arg1 was down-regulated compared with model group. GART, PAICS, and ATIC which participated in purine metabolism decreased significantly in Shikonin-treated groups (Fig. 11). In general, the expression of genes analyzed exhibited high consistency with the results from proteomic analysis.

## Discussion

Colorectal cancer (CRC) is a great menace to the patients who diagnosed with late-stage disease suffering from poor prognosis, especially the elderly generation. In recent years, multiple alternative chemotherapies have been continuously explored to increase survival rates^12^. Shikonin remains one of the most effective chemotherapy agents against various types of cancer in vitro and vivo^13^. Prior study has shown that Shikonin could inhibit the proinflammatory milieu generated at the early phase of colon cancer^14^, induce CRC cells apoptosis and autophagy through JNK signaling pathway^15^, and inhibit the metastatic progression in CRC via up-regulating the expression of SIRT2^16^. However, these studies were mainly limited to reveal molecular targets in traditional pathways, which were hard to interpret the pharmacological mechanisms thoroughly. Therefore, to illustrate the therapeutic mechanism of Shikonin from a systemic perspective, proteomics and metabolomics profile was integrated to investigate the alterations of protein and metabolites in the PDX mice.

In the current study, Shikonin was confirmed to suppress the tumor growth of PDX colon cancer mice in a dose-dependent manner (Fig. 2). Meanwhile, the liver and kidney dysfunction in tumor xenograft mice were alleviated to the normal level after Shikonin treatment compared with the model group (Fig. 3). Furthermore, the potential mechanisms underlying the antitumor effect of Shikonin were explored based on proteomics and metabolomics analysis, which provided insights into global perturbation under Shikonin treatment. A total of 256 DEPs, 32 DEMs in mice tumor tissue, and 20 DEMs in mice serum were identified compared with the model group. The integrated results revealed that the altered pathways were predominantly related to arginine biosynthesis, purine metabolism, biosynthesis of amino acids, and glutathione metabolism (Fig. 10).

Arginine, a semi-essential amino acid, can be synthesized de novo and direct uptake from the bloodstream in normal cells. However, because of the significant disparities between normal cells and cancer cells in metabolism, several types of tumors merely depend on extracellular arginine to support cell growth and survival^17^. Arginine is essential for protein synthesis in adult mammals and plays multiple functions in cell activities. Based on the metabolic vulnerability in tumors, arginine deprivation therapies were developed recently through giving availability to arginine degrading enzymes^18^. In the present study, arginine in mice serum was down-regulated suggesting that the impairment in arginine synthesis and a deficit of exogenous arginine were possibly induced by Shikonin and further inhibit the viability of tumor cells. Moreover, biosynthesis of arginine under physiological conditions was mediated by urea cycle enzymes^19^. The expression of Carbamoyl phosphate synthetase 1 (CPS1) and Ornithine Carbamoyltransferase (OTC), two main enzymes involved in the urea cycle, increased in Shikonin-treated groups. The overexpression of CPS1 and OTC was reported to promote tumor growth by supporting pyrimidine synthesis in some cancer types^20^, while a reversible reduction of the urea cycle enzymes involved in the detoxification of ammonia may further contribute to the development of scar tissue and increase the risk of disease progression^21^. Through activating the urea cycle, the clearance of ammonia was promoted by Shikonin to protect the system function.

More than that, alternative metabolism of purine nucleotides was observed after Shikonin treatment. The salvage pathway and de novo biosynthetic pathway are the two main pathways for purine nucleotides synthesis^22^. Generally, the complementary salvage pathway could satisfy the cellular requirements for purine. However, to satisfy the higher requirements of purine nucleotides for tumor growth, the de novo purine biosynthesis pathway is significantly activated to replenish the purine pool for duplication of genetic materials^23^. The higher levels of xanthine and hypoxanthine in Shikonin-treated groups were sufficient for cell proliferation which reversely implied the decreased requirement for tumor growth. Moreover, Glycinamide ribonucleotide transformylase (GART), a significant trifunctional enzyme participating in de novo purine synthesis decreased in Shikonin-treated groups. Serving as a core enzyme in the nucleotide metabolism, the inhibitor of GART was demonstrated to exert a cytotoxic and cytostatic effect on various types of cancer cell lines^24^. In addition, increased expression of PAICS proved to relate with proliferation, migration, growth, and invasion of CRC cells^25^. The mRNA expression of GART, phosphoribosyl pyrophosphate amidotransferase (PPAT), and phosphoribosylaminoimidazole carboxylase and phosphoribosylaminoimidazolesuccinocarboxamide synthase (PAICS) participating in de novo purine metabolism were down-regulated in Shikonin-treated groups as well. In conclusion, the perturbation above indicated that Shikonin might realize its antitumor effect on colon cancer mainly related to the systemic effect on purine nucleotides synthesis.

The de novo biosynthetic pathway is energy-intensive. To provide sufficient nucleotides for rapid proliferating tumor cells, multitudinous amino acid substrates, and one-carbon units contribute to biosynthetic requirement of purines^26^. The alternation of several amino acids in Shikonin-treated group was observed, such as tyrosine, tryptophan, leucine, and glutamine. These amino acids played a vital role in biotransformation process to supply precursor materials and were identified as metabolic biomarkers of early-stage CRC^27^. Moreover, the decrease of serum tryptophan in patients with CRC was associated with immune suppression^28^. The level of serum tryptophan was detected an increase in Shikonin-treated groups, which indicated the therapeutic mechanism may contribute to immune activation and the inhibition of tumor immune escape. Glutamine is increased to fuel anabolic processes to support cancer growth^29^, the decreasing tendency of glutamine was detected revealing that the inhibition of tumor growth probably induced by Shikonin. In summary, the alteration above provided an expected systemic view of perturbation of arginine biosynthesis, purine metabolism, and biosynthesis of amino acids that get involved in the therapeutic mechanism of Shikonin in colon cancer.

## Conclusion

Taken together, the present study demonstrated the antitumor effect mediated by Shikonin on PDX tumor mice. Meanwhile, proteomics and metabolomics analysis based on the UPLC-MS platform were performed to uncover the global responses to Shikonin treatment. Results suggested that arginine biosynthesis, purine metabolism, and biosynthesis of amino acids were mainly involved in therapeutic mechanism of Shikonin. Altogether, this work provides new insights into exploring the potential mechanisms of the drug effect and might be valuable for further studies on Shikonin mechanism in the clinical treatment of colorectal cancer.

## Materials and methods

### Materials and reagents

HPLC grade methanol (MeOH) and acetonitrile (ACN) were purchased from TEDIA Company Inc. (Fairfield, USA); formic acid was purchased from Sigma-Aldrich (Missouri, USA); ultrapure water was collected using a Milli-Q water purification system (Millipore Corporation, MA, USA). All other reagents and chemicals used in the study were of analytical grade. The assay kits for anine-aminotransferase (ALT), aspartate aminotransferase (AST), serum creatinine (Scr) and blood urea nitrogen (BUN) were purchased from Nanjing Senberga Biotechnology Co., Ltd. (Nanjing, China). Shikonin (purity above 98%) was obtained from Aladdin Biotechnology Co., Ltd. (Shanghai, China) and was dissolved in dimethyl sulfoxide (DMSO) as a stock solution. Shikonin were then diluted in dissolved in 0.5% CMC-Na to concentrations of 0.1 mg/ml and 0.2 mg/ml. The final concentrations of DMSO were did controlled not exceed 0.02%.

### Colon cancer PDX models and animal experiments

The tumor specimen of colon cancer was collected from a patient who had undergone complete surgical resections at the Zhejiang Cancer Hospital colorectal surgery department in March 18, 2019 under Zhejiang Cancer Hospital IRB protocol (Pro002016(87)). Prior written informed consent was obtained from the patient and the experiment was approved by the Zhejiang Cancer Hospital ethics committee (license number: zjzlyy-IRB-2020–1). All studies involving mice were approved by Institutional Animal Care and Use Committee of Zhejiang Cancer Hospital (license number: SCXK (SU) 2017–0,005), and all experiments were performed in accordance with guidelines and regulations approved by Zhejiang Cancer Hospital. No tissue was procured from prisoners and any chemotherapy or radiotherapy prior to surgery were not received by the patient with colon cancer and histology was confirmed by a pathologist from Zhejiang Cancer Hospital. Female nude mice, 5 weeks old, were purchased from the HangSi Biotechnology Co., Ltd. (Hangzhou, China). The mice were housed, fed, and maintained following the recommendations. Colon cancer tissues were implanted into the back of the neck of each mouse after cutting into pieces. A total of 40 animals were divided into 4 groups as follows: 1) control group without tumor grafting 2) model group 3) 1 mg/kg of Shikonin-treated group and 4) 2 mg/kg of Shikonin-treated group. All mice were administered via intraperitoneal injection once every two days for 15 days when the tumors reached a mean of 3 mm × 3 mm. Mice in the control group and model group received the same volume of 0.5% CMC-Na every two days for 15 days. The tumor volume was calculated using the following formula: tumor volume (mm^3^) = (length × width × width). At the final of the experiment, the serum samples from each group were collected via the retroorbital venous plexus and stored at -20 °C for biochemical analysis. The tumor masses were dissected, weighed, and rapidly quenched in liquid nitrogen and stored at -80 °C immediately before analysis.

### Biochemical analysis

The serum was divided into several aliquots to avoid freezing and thawing. Serum levels of alanine aminotransferase (ALT), aspartate aminotransferase (AST), serum creatinine (Scr) and blood urea nitrogen (BUN) were measured according to manufacturer’s prescripts of ELISA kits.

### Sample collection and preparation for proteomics analysis

Frozen tumor tissues were transferred into 1.5 ml tubes and lysed with 500 µL digestion buffer (1 mM PMSF included). The tissue samples were then homogenized on the ice and further lysed with sonication. After that, the samples were subsequently centrifuged at 15,000 g for 15 min at 4 °C to remove insoluble particles and precipitation. Protein concentration was evaluated by Bicinchoninic Acid assay and then store at -80 °C.

The 10 ug proteins of each sample were acquired and separated on 12% sodium dodecyl sulphate polyacrylamide gel electrophoresis (SDS-PAGE) gel. The protein suspensions were then digested with 3 μL sequencing-grade trypsin (1 μg/μL) in 100 μL 300 mM tetraethylammonium bromide (TEAB) buffer and incubated for digestion for 12 h at 37 °C. After that, 40 μL of each sample were transferred into new tubes for TMT labeling. The TMT reagent were added with 88 μL acetonitrile at room temperature and mixed for centrifugation. Then 41 μL of the TMT label reagent was added to each sample. After mixing and being incubated at room temperature for 1 h, 8 µL 5% hydroxylamine were added to each sample and incubated for 15 min to terminate reaction. TMT labeled peptides were separated on an 1,100 HPLC System (Agilent, Technologies, CA, USA).

### Chromatography and mass spectrometry conditions

LC–MS/MS analysis was performed on a TripleTOF 5,600 mass spectrometer (SCIEX, USA) coupled with a Nano spray III source (SCIEX, USA) for 60 min. A capillary C18 trap column (3 cm × 100 µm) following with a C18 column (15 cm × 75 µm) on an E ksigent nanoLC-1D plus system (SCIEX, USA) was utilized for separation. The mobile phase was made of solvent A (0.1% formic acid in water) and solvent B (ACN-H2O-FA, 80: 19.9: 0.1, v/v/v) and remained a constant flow rate at 300 nL/min. Linear gradient was adjusted as follows: 0–45 min, 5–26% B; 45–52 min, 26–60% B; 52–53 min, 60–95% B; 53–60 min, 95% B.

The mass spectrometer was performed both in positive ion mode, and parameters were established as follows: spray voltage: 2.5 kV ( +); automatic gain control (AGC) target: 2 × 10^5^ ions; centroid mass data with full scan: m/z 350–1950; MS/MS mode collision energy: 38 eV; peptide detection resolution of MS/MS: 15,000; automatic gain control (AGC) target: 5 × 10^4^ ions; maximum ion injection time (IT): 100 ms; dynamical exclusion time for Mass was set at 60 s.

### Protein identification and pathway analysis

The MS/MS spectra were analyzed for protein identification and quantification using Proteome Discoverer 2.4 (Thermo Fisher Scientific, MA, USA). Only proteins with global FDR ≤ 1% and unique peptides ≥ 1 were considered for further downstream analysis. Proteins which screened with fold change > 1.2 (or < 0.8) and *p* value < 0.05 were selected as differentially expressed proteins (DEPs). Meanwhile, biological function analysis of the DEPs was performed via the DAVID 6.8 (https://david.ncifcrf.gov). The KEGG pathway^30,31^ and GO analysis (cellular components, molecular functions and biological processes) were included. The protein–protein interaction network (PPI) was constructed utilizing the STRING database (https://string-db.org).

### Sample collection and preparation for metabolomics analysis

An aliquot of 20 mg of tumor tissues was weighted and 400 µL cold methanol was added for metabolites extraction. After being ground with breads for 3 min and then centrifuged at 13,000 rpm for 15 min at 4 °C, the supernatants were transferred to the new 1.2 mL polypropylene tubes and 400 µL cold water then added, the mixture was subsequently frozen in -80 °C refrigerator for 15 min. The frozen samples were dried in the freeze dryer immediately for 3 h. The residues were re-dissolved in 80µL acetonitrile (ACN)/H2O (20:80, v/v) and centrifuged at 13,000 rpm for 15 min at 4 °C. An aliquot of 60µL supernatant of each sample was then transferred to vials for UPLC-MS analysis in a random order.

Serum sample (50 μl) of each mouse was collected for metabolomic experiments. Acetonitrile (150 μl) was added for precipitating the proteins and then vortexed for 30 s. After centrifuging at 13,000 rpm for 15 min at 4 °C, the supernatants were transferred to the new 1.2 mL polypropylene tubes and then frozen in -80 °C refrigerator for 15 min. The frozen samples were dried in the freeze dryer immediately for 3 h. The residues were re-dissolved in 80µL ACN/H_2_O (20:80, v/v) and centrifuged at 13,000 rpm for 15 min at 4 °C. An aliquot of 60µL supernatant of each sample was then transferred to vials for UPLC-MS analysis in a random order.

A quality control (QC) sample was prepared by mixing an equal aliquot (40 μL) from all tissue samples and serum samples for the optimization of the UPLC-MS conditions, and the method validation.

### Chromatography and mass spectrometry conditions

An Ultimate 3,000 UHPLC system coupled with a Q Exactive Hybrid Quadrupole-Orbitrap Mass Spectrometer (Thermo Scientific, Germany) was used for metabolomic analysis. ACQUITY UPLC HSS T3 column (2.1 mm × 100 mm × 1.8 µm, Waters, USA) was applied for metabolites separation at 40 °C. The 0.1% formic acid in water (solvent A) and acetonitrile (solvent B) was performed and a constant flow rate was remained at 0.3 mL/min. The autosampler temperature was set at 4 °C. The gradient elution was adjusted as follows: 0–1 min: 2% B, 1–12 min: 2–100% B, 12–15 min: 100% B, 15–16 min: 2% B. The injection volume of each sample for analysis is 5 µL. Quality control (QC) samples were performed every 10 samples to ensure system stability and repeatability. Mass spectrometry was performed in both positive and negative mode (ESI), and parameters were set as follows: spray voltage: 3.5 kV (+) and 2.5 kV (−); capillary temperature: at 350 °C (+) and 320 °C (−); sheath gas (N_2_) flow rate: 35 arb (+) and 40 arb (−); auxiliary gas (N_2_) flow rate: 10 arb (+) and 8 arb (−); probe heater temperature : 320 °C (+) and 350 °C (−); S-Lens RF level: 55; automatic gain control (AGC) target: 1 × 10^6^ ions; maximum ion injection time (IT): 100 ms; peptide detection resolution: 70,000; centroid mass data with full scan: m/z 70–1,000; MS/MS mode was set at three collision energy: 10, 20 and 40 eV.

### Multivariate data analysis

Raw data from UPLC-MS were converted into mzXML format through MSconvert tool (https://proteowizard.sourceforge.net/downloads.shhtml) for further analysis. The R package XCMS (v3.4.1) was used for nonlinear retention time correction, peak filtration and extraction. Subsequently, the profile containing mass to charge ratio (m/z), retention time and ion intensity were further processed by MetaX package of R (v3.4.1). In which the signal correction and peak normalization were performed according to the quality control samples (QC). Metabolites with coefficient of variation (CV) value > 30% in QC samples were excluded for the metabolite’s discovery. Batch normalization of peak area was applied to compare the data from different samples and multivariate statistical analysis was performed by SIMCA-P 14.1 software (Umetrics, Sweden) using unit variance scaling and mean-centered method. Principal Component Analysis (PCA) and Orthogonal Partial Least Square Discriminant Analysis (OPLS-DA) were applied to discriminate control and drug-treated group. Metabolites which changed significantly among different groups were screened with variable importance in the projection (VIP) exceeding 1.2 and ANOVA *p* value < 0.05 were finally identified as differentially expressed metabolites (DEMs). Both METLIN (https://metlin.scripps.edu) and HMDB (https://www.hmdb.ca/) were applied for the identification of filtered metabolites. And part of differential metabolites was further confirmed by matching both MS/MS spectra and the retention time with commercially available standards. Heatmap package in R(v3.4.1) was used to further determine the metabolic patterns of DEMs among all groups. Meanwhile, the relevant metabolic pathways were enriched by MetaboAnalyst 4.0 (https://www.metaboanalyst.ca) as well to discover the significant pathways regulated by Shikonin.

### Real-time quantitative PCR

Total RNA was extracted from culture cells by Trizol reagent, and PrimeScript II 1st Strand cDNA Synthesis Kit (TaKaRa, Japan) was used to synthesize cDNA with mRNA-specific primers. The primers for differentially expressed mRNAs and control GAPDH were obtained from Proteintech (Rosemont, USA). The real-time quantitative PCR (RT-qPCR) was performed on Applied Biosystems 7,500 Real-Time PCR machine. The 2^-CT^ method was used to determine the mRNAs relative expression and all reactions were repeated three times. The sequences of primers used are listed in Table S3.

### Statistical analysis

The experimental results were presented as mean ± SD. Statistical analysis was performed by the two-tailed unpaired student’s t-test or one-way ANOVA followed by Tukey’s multiple comparison test using SPSS software (version 20.0, SPSS, Chicago, IL, USA). Differences with a *p* value < 0.05 were considered significant.

## Supplementary information

Supplementary file1
