# Supplementary material for: Integrated proteomics and metabolomics reveals the comprehensive characterization of antitumor mechanism underlying Shikonin on colon cancer patient-derived xenograft model
Source: Sci Rep. 2020 Aug 24;10:14092. doi: 10.1038/s41598-020-71116-5 (PMC7445290; doi:10.1038/s41598-020-71116-5)
Supplement: Supplementary file 1 — Supplementary file1 [file 41598_2020_71116_MOESM1_ESM.docx]

Supporting Information for “**Integrated Proteomics and Metabolomics Reveals the Comprehensive Characterization of Antitumor Mechanism Underlying Shikonin on Colon Cancer Patient-Derived Xenograft Model**”

**Yang Chen^1^, Juan Ni^2,3,4^, Yun Gao^2,3,4^, Jinghui Zhang^1^, Xuesong Liu^1^, Yong Chen^1^, Zhongjian Chen^2,3,4,^ *, and Yongjiang Wu^1,^ ***

^1^ College of Pharmaceutical Sciences, Zhejiang University, Hangzhou 310058, China;

^2^ Institute of Cancer and Basic Medicine (ICBM), Chinese Academy of Sciences, Hangzhou, Zhejiang Province, 310022, China;

^3^ Cancer Hospital of the University of Chinese Academy of Sciences, Hangzhou, Zhejiang Province, 310022, China;

^4^ Zhejiang Cancer Hospital, Hangzhou, Zhejiang Province, 310022, China;

^*^ Correspondence:

**Contents:**

**Figures**

**Figure S1** Heatmap and PPI networks analysis of 13 DEPs involved in high-dosage of Shikonin and model groups.

**Tables**

**Table S1** Diﬀerentially expressed metabolites (DEMs) from PDX mice tumor tissue metabolic analysis.

**Table S2** Diﬀerentially expressed metabolites (DEMs) from PDX mice serum metabolic analysis.

**Table S3** Primers sequence for RT-qPCR


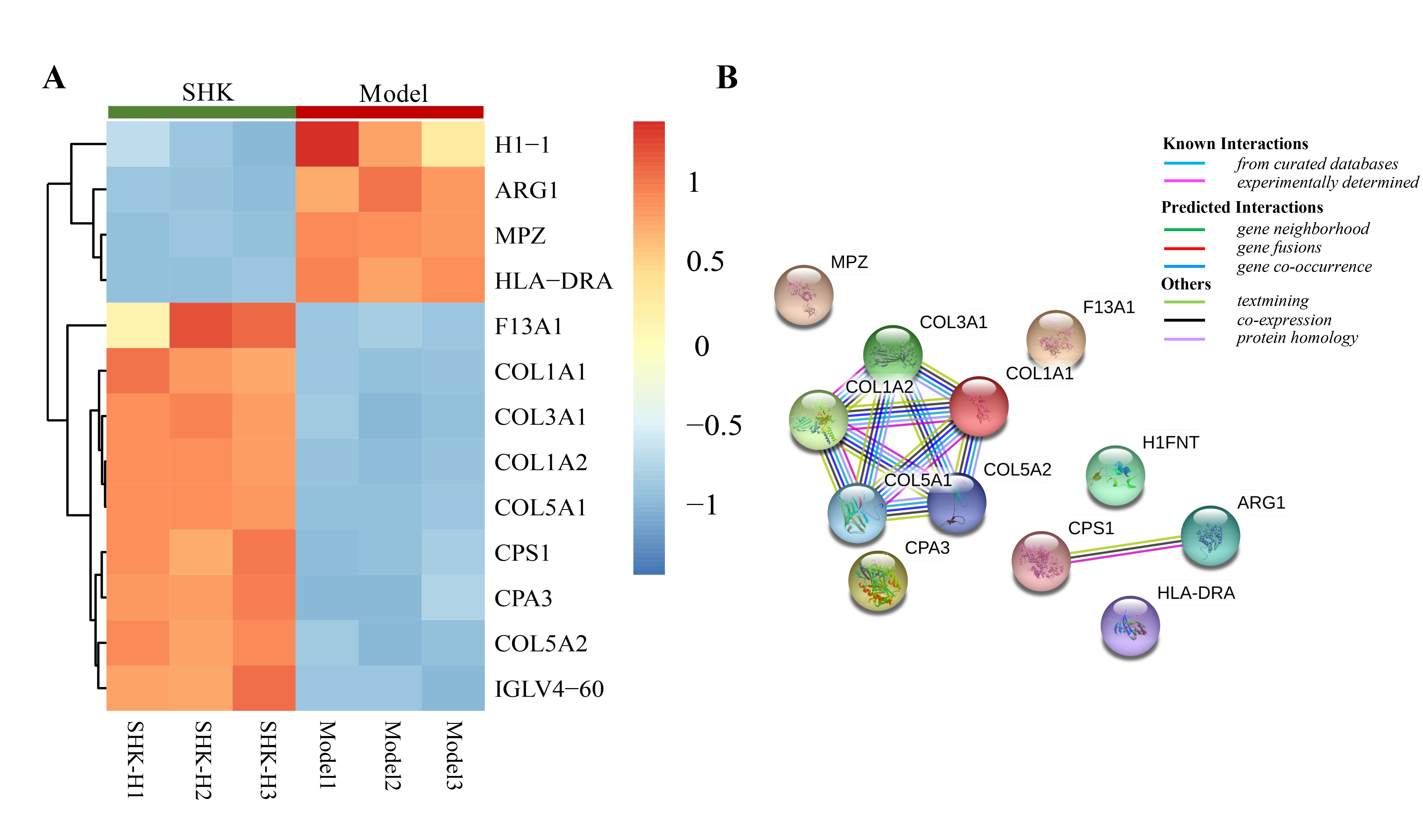


**Figure S1.** Heatmap and PPI networks analysis of 13 DEPs involved in high-dosage of Shikonin and model groups.

**Table S1.** Diﬀerentially expressed metabolites (DEMs) from PDX mice tumor tissue metabolic analysis.

| Mode | No. | Metabolite | Measured m/z | RT/min | VIP^a^ | *P*-value^b^ | SHK-L vs Model | |  | SHK-H vs Model | |
| --- | --- | --- | --- | --- | --- | --- | --- | --- | --- | --- | --- |
|  |  |  |  |  |  |  | FC^c^ | FDR^d^ |  | FC^c^ | FDR^d^ |
| ESI+ | T1 | L-Leucine | 132.10139 | 2.19 | 19.23 | 4.62E-03 | 0.84 | 4.30E-02 |  | 1.49 | 2.30E-02 |
|  | T2 | Niacinamide | 123.05497 | 1.52 | 16.74 | 5.56E-04 | 1.10 | 4.40E-01 |  | 2.21 | 3.20E-04 |
|  | T3 | L-2-Aminoadipic acid | 162.07528 | 1.12 | 11.26 | 8.59E-04 | 0.87 | 2.35E-01 |  | 0.61 | 6.10E-04 |
|  | T4 | L-Tyrosine | 182.08043 | 2.04 | 10.18 | 3.55E-04 | 1.09 | 5.80E-01 |  | 2.37 | 1.30E-04 |
|  | T5 | Hypoxanthine | 137.04524 | 3.15 | 7.59 | 1.15E-04 | 1.64 | 2.10E-03 |  | 2.93 | 2.20E-05 |
|  | T6 | Propionylcarnitine | 218.13774 | 2.76 | 6.03 | 3.45E-04 | 1.38 | 5.27E-01 |  | 1.97 | 3.60E-04 |
|  | T7 | 4-Hydroxycinnamic acid | 165.05395 | 2.04 | 4.29 | 2.44E-04 | 1.10 | 4.80E-01 |  | 2.51 | 1.30E-04 |
|  | T8 | L-Proline | 116.07044 | 1.15 | 3.52 | 5.42E-04 | 0.93 | 2.62E-01 |  | 0.75 | 4.02E-04 |
|  | T9 | D-Glutamic acid | 130.04943 | 1.14 | 3.50 | 3.14E-03 | 1.09 | 2.50E-01 |  | 0.71 | 1.50E-03 |
|  | T10 | Phosphocholine | 184.07251 | 0.89 | 2.96 | 4.82E-03 | 0.91 | 7.16E-01 |  | 0.64 | 5.11E-03 |
|  | T11 | L-Glutamine | 147.07576 | 0.89 | 2.74 | 2.03E-02 | 0.90 | 9.10E-01 |  | 0.62 | 8.90E-02 |
|  | T12 | Adenosine | 268.10279 | 2.80 | 2.51 | 5.09E-03 | 0.88 | 6.38E-01 |  | 1.31 | 4.23E-02 |
|  | T13 | Choline | 104.10702 | 1.19 | 2.45 | 6.50E-02 | 1.15 | 1.90E-01 |  | 1.47 | 2.90E-02 |
|  | T14 | Adenine | 136.06109 | 1.16 | 1.96 | 3.41E-03 | 1.26 | 2.90E-03 |  | 1.14 | 1.50E-02 |
|  | T15 | L-Alanine | 90.05520 | 4.22 | 1.88 | 3.55E-02 | 1.41 | 1.50E-02 |  | 1.17 | 1.10E-01 |
|  | T16 | Phenylpropiolic acid | 147.04340 | 2.03 | 1.75 | 2.50E-04 | 1.12 | 5.30E-01 |  | 2.50 | 1.30E-04 |
| ESI-  ESI- | T17 | Inosine | 267.07428 | 3.16 | 8.53 | 8.96E-04 | 1.75 | 2.90E-02 |  | 2.65 | 4.30E-05 |
|  | T18 | Xanthine | 151.02533 | 1.84 | 7.67 | 6.80E-04 | 1.43 | 6.30E-02 |  | 2.49 | 7.60E-05 |
|  | T19 | Oxidized glutathione | 611.14705 | 2.05 | 6.17 | 4.30E-02 | 0.99 | 8.50E-01 |  | 1.55 | 4.30E-03 |
|  | T20 | N-Acetylneuraminic Acid | 308.09974 | 0.93 | 3.98 | 1.98E-03 | 1.14 | 4.11E-01 |  | 0.73 | 3.76E-02 |
|  | T21 | Uridine | 243.06271 | 1.94 | 3.67 | 1.08E-03 | 1.20 | 7.50E-02 |  | 2.03 | 2.10E-04 |
|  | T22 | Fumaric acid | 115.00257 | 1.73 | 3.23 | 2.16E-02 | 1.07 | 9.64E-01 |  | 1.48 | 3.08E-02 |
|  | T23 | 2-Keto-glutaramic acid | 289.06870 | 1.95 | 3.18 | 1.08E-02 | 1.27 | 1.50E-02 |  | 1.99 | 1.50E-02 |
|  | T24 | 3-Dehydroquinic acid | 189.04012 | 2.01 | 3.00 | 6.17E-03 | 1.32 | 5.20E-02 |  | 1.75 | 1.50E-03 |
|  | T25 | myo-Inositol | 179.05562 | 0.94 | 2.36 | 1.11E-02 | 0.91 | 2.17E-01 |  | 0.78 | 8.07E-03 |
|  | T26 | Indoxylsulfuric acid | 212.00231 | 5.13 | 2.18 | 7.02E-04 | 0.97 | 6.30E-01 |  | 0.43 | 2.10E-04 |
|  | T27 | 4-Hydroxybenzaldehyde | 121.02844 | 5.58 | 2.06 | 1.32E-05 | 1.54 | 1.61E-05 |  | 1.41 | 4.54E-04 |
|  | T28 | ADP | 426.02393 | 1.02 | 1.47 | 1.03E-02 | 0.84 | 3.69E-01 |  | 0.60 | 7.68E-03 |
|  | T29 | 2-keto valeric acid | 115.03895 | 3.22 | 1.42 | 1.93E-02 | 0.62 | 6.30E-02 |  | 0.48 | 6.80E-03 |
|  | T30 | N-Acetyl-L-methionine | 190.05403 | 4.78 | 1.35 | 3.81E-02 | 1.30 | 1.70E-01 |  | 1.38 | 3.46E-02 |
|  | T31 | Aminomalonic acid | 118.01481 | 12.36 | 1.34 | 5.97E-03 | 0.83 | 6.30E-01 |  | 0.60 | 2.90E-03 |
|  | T32 | Hippuric acid | 178.05051 | 5.21 | 1.23 | 9.37E-04 | 0.73 | 1.20E-01 |  | 0.33 | 7.60E-05 |

^a^ The VIP value was obtained from OPLS-DA model with a threshold of 1.2.

^b^ *P*-value from one-way ANOVA test.

^c^ FC was calculated from comparing low-dosage Shikonin treatment *vs* model group or high-dosage Shikonin treatment *vs* model group.

^d^ Value of FDR was obtained from the adjusted T-test P value of FDR correction by Benjamini-Hochberg method.

**Table S2.** Diﬀerentially expressed metabolites (DEMs) from PDX mice serum metabolic analysis.

| Mode | No. | Metabolite | Measured m/z | RT/min | VIP^a^ | *P*-value^b^ | SHK-L vs Model | |  | SHK-H vs Model | |
| --- | --- | --- | --- | --- | --- | --- | --- | --- | --- | --- | --- |
|  |  |  |  |  |  |  | FC^c^ | FDR^d^ |  | FC^c^ | FDR^d^ |
| ESI+ | S1 | Xanthine | 153.04004 | 3.83 | 2.15 | 2.46E-03 | 0.32 | 1.90E-02 |  | 0.69 | 4.30E-02 |
|  | S2 | L-Acetylcarnitine | 204.12217 | 3.62 | 2.12 | 5.51E-05 | 1.08 | 7.71E-01 |  | 0.52 | 6.20E-04 |
|  | S3 | L-Arginine | 175.11816 | 0.86 | 1.51 | 1.37E-02 | 1.40 | 1.76E-01 |  | 0.87 | 1.27E-04 |
|  | S4 | Choline | 104.10700 | 8.72 | 1.43 | 1.94E-02 | 0.95 | 6.33E-02 |  | 0.71 | 2.36E-02 |
|  | S5 | Ureidopropionic acid | 115.05004 | 1.46 | 1.30 | 2.35E-04 | 1.49 | 2.59E-03 |  | 1.59 | 3.33E-04 |
|  | S6 | Phosphohydroxypyruvic acid | 184.98473 | 12.73 | 1.28 | 3.11E-03 | 0.94 | 8.58E-01 |  | 0.60 | 4.30E-03 |
|  | S7 | alpha-Ketoisovaleric acid | 99.04418 | 4.02 | 1.24 | 4.22E-03 | 0.69 | 1.10E-02 |  | 0.62 | 1.50E-03 |
|  | S8 | Indoleacetaldehyde | 160.07501 | 6.01 | 1.22 | 2.73E-02 | 1.00 | 9.10E-01 |  | 1.77 | 2.90E-02 |
| ESI- | S9 | Hippuric acid | 178.05044 | 5.19 | 3.90 | 3.55E-04 | 1.37 | 5.30E-01 |  | 0.31 | 2.10E-04 |
|  | S10 | Oxoglutaric acid | 145.01334 | 1.21 | 3.16 | 8.39E-03 | 1.50 | 2.10E-01 |  | 1.97 | 5.99E-03 |
|  | S11 | Oxalosuccinic acid | 189.00237 | 5.71 | 2.99 | 4.07E-04 | 0.94 | 5.30E-01 |  | 0.10 | 4.90E-04 |
|  | S12 | 2-Hydroxyisocaproic acid | 131.07034 | 5.42 | 2.81 | 7.28E-04 | 0.95 | 8.37E-01 |  | 0.66 | 1.13E-03 |
|  | S13 | Caffeic Acid | 179.03449 | 4.20 | 2.78 | 3.36E-04 | 1.66 | 2.90E-02 |  | 4.17 | 1.30E-04 |
|  | S14 | Hypoxanthine | 135.03025 | 1.69 | 2.75 | 2.65E-02 | 0.50 | 2.26E-02 |  | 0.67 | 1.60E-02 |
|  | S15 | Xanthosine | 283.06922 | 3.83 | 2.72 | 3.73E-03 | 0.39 | 2.30E-02 |  | 0.92 | 4.20E-02 |
|  | S16 | Uridine | 243.06268 | 2.08 | 2.56 | 2.40E-04 | 1.91 | 1.91E-04 |  | 1.61 | 1.03E-02 |
|  | S17 | L-Tryptophan | 203.08237 | 4.55 | 2.25 | 8.02E-03 | 1.25 | 3.44E-01 |  | 1.59 | 5.91E-03 |
|  | S18 | Dehydroascorbic acid | 173.00865 | 1.07 | 2.06 | 1.82E-04 | 1.60 | 1.76E-01 |  | 2.59 | 1.27E-04 |
|  | S19 | 4-Pyridoxic acid | 182.04540 | 3.22 | 1.82 | 1.21E-03 | 1.06 | 8.51E-01 |  | 0.61 | 7.20E-03 |
|  | S20 | Cysteinyl-Aspartate | 217.02985 | 1.26 | 1.38 | 6.94E-04 | 1.88 | 7.60E-05 |  | 1.62 | 2.30E-02 |

^a^ The VIP value was obtained from OPLS-DA model with a threshold of 1.2.

^b^ *P*-value from one-way ANOVA test.

^c^ FC was calculated from comparing low-dosage Shikonin treatment *vs* model group or high-dosage Shikonin treatment *vs* model group.

^d^ Value of FDR was obtained from the adjusted T-test P value of FDR correction by Benjamini-Hochberg method

**Table S3.** Primers sequence for RT-qPCR

| **Genes** | **Forward** | **Reverse** |
| --- | --- | --- |
| CPS1 | TTGGCAAGAAGACAGTGGTG | GAGGGCTTGTACCCATGATC |
| OTC | GCTCAACAATGCAGCTCTTAG | GTGGACAGTCTTGTTCGAGTAC |
| Arg1 | CAGAAGAATGGAAGAGTCAG | CAGATATGCAGGGAGTCACC |
| GART | CCATCTAAGGTGGACTGA | TGCTGGAATAATGCTGAC |
| PAICS | AAACAGAATGCTCCCAACT | AACCCAAGAACCAATGAA |
| ATIC | GTGGTGGAAGAGCCGAAG | GCAGGAGATAAGGCAAAC |
| GAPDH | GAAGGTGAAGGTCGGAGT | GAAGATGGTGATGGGA |
